# Circadian Alterations in a Murine Model of Hypothalamic Glioma

**DOI:** 10.3389/fphys.2017.00864

**Published:** 2017-10-30

**Authors:** José M. Duhart, Lucila Brocardo, Carlos S. Caldart, Luciano Marpegan, Diego A. Golombek

**Affiliations:** Laboratorio de Cronobiología, Departamento de Ciencia y Tecnología, Universidad Nacional de Quilmes, Buenos Aires, Argentina

**Keywords:** circadian, suprachiasmatic, glioma, glioblastoma

## Abstract

The mammalian circadian system is controlled by a central oscillator located in the suprachiasmatic nuclei (SCN) of the hypothalamus, in which glia appears to play a prominent role. Gliomas originate from glial cells and are the primary brain tumors with the highest incidence and mortality. Optic pathway/hypothalamic gliomas account for 4–7% of all pediatric intracranial tumors. Given the anatomical location, which compromises both the circadian pacemaker and its photic input pathway, we decided to study whether the presence of gliomas in the hypothalamic region could alter circadian behavioral outputs. Athymic nude mice implanted with LN229 human glioma cells showed an increase in the endogenous period of the circadian clock, which was also less robust in terms of sustaining the free running period throughout 2 weeks of screening. We also found that implanted mice showed a slower resynchronization rate after an abrupt 6 h advance of the light-dark (LD) cycle, advanced phase angle, and a decreased direct effect of light in general activity (masking), indicating that hypothalamic tumors could also affect photic sensitivity of the circadian clock. Our work suggests that hypothalamic gliomas have a clear impact both on the endogenous pacemaking of the circadian system, as well as on the photic synchronization of the clock. These findings strongly suggest that the observation of altered circadian parameters in patients might be of relevance for glioma diagnosis.

## Introduction

Gliomas, a type of tumor arising from glial cells, are the most common type of primary brain tumors and are classified in four types (I-IV), according to their malignancy (Wen and Kesari, 2008). Patients suffering from type IV gliomas (also characterized as glioblastomas) account for approximately 45% of all malignant CNS tumors, with a median survival of less than 15 months (Ostrom et al., 2013; Lee, 2017). Among the common symptoms leading to glioma diagnosis, neurological distress appears as one of the earliest reasons for consultation (Weller et al., 2017). Previous reports have shown that two of the most common factors affecting the life quality of glioma patients are sleep disturbances and fatigue (Rooney et al., 2013). Brain and CNS tumors are the most common form of solid tumors in children and among them, anatomical location of hypothalamic and optic tract tumors represent 5–7% of all cases (Massimi et al., 2007; Ostrom et al., 2013). These gliomas tend to be nonaggressive low-grade tumors, with pilocytic astrocytoma being the most common pathology in young adults. However, chiasmatic-hypothalamic masses in adults are challenging to diagnose and tend to have an aggressive clinical course (Raelson and Chiang, 2015).

The hypothalamus also hosts the mammalian circadian clock, led by a master oscillator that resides in the suprachiasmatic nuclei (SCN), which receives photic entrainment cues through a retinohypothalamic tract. This clock is able to sustain oscillations with a period close to 24-h, even in the absence of external stimulation, and it can adapt to changes in the light-dark cycle through a specific synchronization pathway (Golombek and Rosenstein, 2010). Several physiological and behavioral variables, including hormone levels and neurological functions, are regulated by the circadian clock, which also plays a key role in the regulation of sleep-wake cycles (Musiek and Holtzman, 2016). Moreover, circadian disruption and misalignment between the internal clock and the environmental cycles have been associated with mood disorders and fatigue (Golombek et al., 2013; Bedrosian and Nelson, 2017).

Within the SCN, glial cells are involved in circadian timekeeping (Barca-Mayo et al., 2017; Brancaccio et al., 2017; Tso et al., 2017) and synchronization mechanisms (Lavialle et al., 2001; Becquet et al., 2008; Girardet et al., 2010; Duhart et al., 2013b), and are also considered as mediators between proinflammatory signals and the circadian pacemaker (Leone et al., 2006; Duhart et al., 2013a). Among the mechanisms proposed for glial modulation of the circadian pacemaker, the regulation of glutamate levels by SCN astrocytes is of key importance for the proper functioning of the clock (Beaulé et al., 2009; Leone et al., 2015; Brancaccio et al., 2017). Noteworthy, increased glutamate levels are characteristic in gliomas (reviewed in Robert and Sontheimer, 2014), suggesting that a dysregulation of proper glial function occurring in this malignant tissue may impact on both timekeeping and synchronization mechanisms of the clock. In addition, molecules involved in immune responses, such as TNF-α, IL-1β, and CCL2 have been shown to affect the master circadian oscillator (Nygård et al., 2009; Leone et al., 2012; Duhart et al., 2016). Gliomas drastically alter the microenvironment in which they develop, and molecules involved in the immune response have been shown to have an important role in tumor progression (Reviewed in Christofides et al., 2015).

Considering the prevalence of sleep alterations and fatigue as a common symptom of gliomas, the anatomical compromise of the master clock and optic tract in hypothalamic gliomas, and the alteration of molecules relevant for the circadian pacemaker in the tumor microenvironment, we hypothesized that this pathology could impact circadian timekeeping, and such alterations could be a valuable tool at the time of diagnosis. In the present work we analyzed the effects of hypothalamic gliomas in the endogenous characteristics of the circadian clock and its ability to synchronize to light. In mice bearing hypothalamic gliomas we found alterations in the synchronization to light-dark cycles, as well as a clear effect on the endogenous period and its stability.

## Materials and methods

### Animals

Adult Foxn1^(Δ/Δ)^ male mice were purchased from Universidad Nacional de La Plata animal facilities (La Plata, Argentina), and were housed individually in cages, supplied with water and food *ad libitum* and kept under sterile air ventilation and 12:12 light-dark cycles (12 h of light, 12 h of darkness, LD). Animal manipulations and experimental protocols performed in this work were supervised and approved by the National University of Quilmes Institutional Animal Care and Use Committee, in accordance with the National Institutes of Health guide for the care and use of laboratory animals. After tumor implantation, animals were monitored daily in search for hunched or abnormal posture, lack of grooming, weight loss exceeding 20% of body weight, anorexia, or abnormal ambulation. The presence of any of these findings was considered an endpoint and mice were euthanized using a CO_2_ chamber.

### Cell lines

Human glioblastoma cell line LN-229 (ATCC: CRL-2611) was cultured in DMEM medium (Gibco, Thermo Fisher Scientific) supplemented with 10% fetal bovine serum (PAA, Germany), glutamine and antibiotic-antimicotic (Gibco).

### Tumor implantation

Two month-old animals were deeply anesthetized with 2% isofluorane and implanted with 200,000 glioma LN-229 cells in 1 μl of DMEM by stereotactic surgery. The injection was aimed unilaterally to the suprachiasmatic nucleus of the hypothalamus (coordinates from Bregma: +0.3 mm from midline; −0.1 mm anterior-posterior; −5.3 mm dorso-ventral). Control animals were treated with a Sham surgery in which the same volume of DMEM, without cells, was injected.

### Behavioral analyses

General activity of animals was continuously registered by infrared sensors with a system designed in our laboratory. Activity counts were collected every 5 min. In LD conditions, time is described as Zeitgeber Time (ZT): ZT0 refers to the time of lights on and ZT12 refers to the time of lights off. In constant darkness (DD) conditions, time is expressed as Circadian Time (CT), with CT 12 referring to the time of activity onset.

Prior to tumor implantation, mice were synchronized to 12:12 LD cycles and kept under these conditions for 25 days. After implantation, animals continued under LD 12:12 cycles for 18 days. Afterwards, animals were divided in two groups. One group, aimed to study the effects on circadian synchronization, was exposed to a 6-h advance in the LD cycle (experimental Jet-lag) by advancing the light phase and shortening of the dark phase. This group continued in LD conditions for 32 days, received a second 6-h advance in the LD cycle and continued in LD until endpoint. The second group, aimed to study the endogenous circadian parameters, was kept in DD conditions until endpoint. After sacrifice, brains were collected and processed for Hematoxilyn-Eosin (HE) staining.

### Data analysis

Circadian parameters were calculated with El Temps program (Antoni Díez Nogueira, University of Barcelona). In DD conditions, free-running endogenous period (τ) was analyzed using Sokolove-Bushell (SB) periodograms. For period stability, a wavelet-based analysis was performed on custom-made Matlab (Mathworks) scripts. Heat maps referring to instant period were generated for each activity pattern, assessing the variability of endogenous period. Statistical significance for periods calculated by wavelet analysis were performed as described in Torrence and Compo (1998). A *variability index (V)* was defined by quantifying the complexity of the histogram of the periods obtained from the wavelet analysis by a Shannon entropy estimation (Masè et al., 2005). We first obtained the instant periods from the wavelet transform and chose a bin size for the histogram construction (in this case we used twice the sample size). To compute the Shannon entropy (SE) of the period distribution we used the following formula:

$$\begin{array}{l} \text{SE}=\;-\overset{m}{\underset{i=i}{\sum }}p\left(i\right)\ln p\left(i\right) \end{array}$$

Where *m* is the number of bins with non-zero probability and *p(i)* is the probability of assuming the *i*th value (frequency of occurrence of *i* in N observations). To relate the dispersion of the period distribution with the stability of the system, the *V* index was defined as follows:

$$\begin{array}{l} V=1-\;\frac{SE}{\ln\;N} \end{array}$$

The *V* index ranges from 0, when the spreading of the period is maximal, to 1, when the probability distribution have a single period.

In addition, Non-Parametric Circadian Rhythm Analysis (NPCRA) was performed to analyze the interdaily stability (IS index), and intradaily variability (IV index) of locomotor activity rhythms in constant darkness (Van Someren et al., 1999). The IS index was defined as the most significant period value from the chi-square periodogram, normalized for the number of data, and can be calculated as the ratio between the variance around the mean of the most significant period and the overall variance:

$$\begin{array}{l} IS=\;\frac{n\sum_{h=1}^{p}{\left({\bar{x}}_{h}-\bar{x}\right)}^{2}}{p\sum_{i=1}^{n}{\left(x_{i}-\bar{x}\right)}^{2}} \end{array}$$

Where *n* is the total number of data, *p* is the number of data per day (according to the period), ${\bar{x}}_{\text{h}}$ are the hourly means, $\bar{x}$ is the mean of all data, and *x*_*i*_ represents the individual data points. The IS tends to zero with Gaussian Noise and 1 for a perfect coupling. The IV index was calculated as the ratio of the mean square of the difference between all the successive hours (first derivate) and the same computation around the overall mean:

$$\begin{array}{l} IV=\;\frac{n\sum_{i=2}^{n}{\left(x_{i}-\;x_{i-1}\right)}^{2}}{\left(n-1\right)\sum_{i=1}^{n}{\left(x_{i}-\bar{x}\right)}^{2}} \end{array}$$

The IV value is zero for a perfect sinusoidal wave, and is around 2 for Noise (Gaussian Noise).

For subjective night duration (α), an hourly waveform was generated for the average activity of 15 days in DD conditions, and time was calculated as time length covered by the portion of the curve surpassing the mean of activity baseline. Conversely, subjective day duration (ρ) was calculated as the time length where the curve was under the mean activity baseline. Activity during α was calculated as the area under the curve during subjective night phase, normalized to total activity.

In LD conditions, 3 stages of the experiment were analyzed: Before-Implantation (BI), Post-Implantation (PI) and Post-jetlag (P-JL). Each period was analyzed separately: BI was the synchronization stage prior to glioma implantation, PI was considered from 4 days after surgery until the phase shift of the cycle, and P-JL stage corresponded to the days after the animals had resynchronized to the new LD cycle, until the end of experiment. Activity onset was calculated using individual waveforms of mean activity as the time at which the activity curve overpassed the mean baseline for more than 2 h. Phase angle was defined as the difference between the time of lights off (ZT 12) and the time of activity onset, with positive values referring to activity onset occurring before lights off. LD period was calculated with SB periodograms. Diurnal activity was calculated as the area under the curve that was below the mean activity baseline of individual waveforms for each stage, normalized to total mean activity. Resynchronization speed was calculated as described in Kiessling et al. (2010). Briefly, PS_50_, which describes the amount of time in days needed to achieve 50% of the resynchronization after the Jet-lag shift, was calculated by adjusting a sigmoid dose-response curve with variable slope to the onset of locomotor activity time points for each group, with the following equation:

$$\begin{array}{l} y=Bottom+\frac{\left(Top-Bottom\right)}{1+10^{\left(\log PS50-x\right)\;.\;HillSlope}} \end{array}$$

Differences between the best-fit PS_50_ values were compared by extra sum-of-squares *F*-test.

In addition, the difference between the phase of activity onset in the last 10 days of LD cycles and the first 10 days in DD conditions (Δψ) was analyzed with circular statistics by Rayleigh tests. First, individual Rayleigh tests for LD and DD conditions were applied for each data set using the onset of activity as a phase (ψ) marker. For LD Rayleigh plots, a 24 h period was used, and for DD Rayleigh plots we applied the endogenous period. For each animal, the average Δψ_LD−DD_ was calculated and used as entry data for a group Rayleigh analysis, using a 24 h period.

Data is presented as Mean ± SEM. Statistical analysis was performed with GraphPad Prism 6 program. *P*-values minor to 0.05 were considered as statistically significant.

## Results

### Hypothalamic glioma model

**Figure 1:**
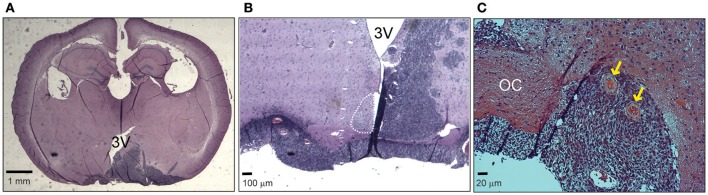
Hypothalamic LN229 glioblastoma cells implantation. **(A)** An example of a coronal slice of brain tissue where implanted cells resulted in tumor masses compromising hypothalamic, ventricular and optic tract structures. **(B)** Another example showing a brain where the tumor affected the ipsilateral SCN and the optic chiasm. **(C)** Tumor implantation resulted in vascularization of the tissue (yellow arrows). OC, Optic chiasm; 3V, third ventricle.

In order to analyze the effects of hypothalamic gliomas on the circadian clock, we performed a unilateral implantation of LN-229 human glioblastoma cells in the region of the SCN. This resulted in 100% implantation efficiency and a mean survival of 54 ± 4 days (*n* = 15). Although we did not find an identical distribution of the tumors among different animals, all of them showed masses compromising the ipsilateral hypothalamus, optic chiasm and part of the third ventricle, as well as vascularization of the tumor (Figure 1).

### Effects of tumor implantation on endogenous circadian timekeeping

The ability of the circadian clock to sustain proper endogenous timekeeping is fundamental in order to adjust an appropriate phase relation of clock-controlled outputs to the environment. To study the implications of hypothalamic gliomas on the endogenous properties of the circadian clock, we analyzed different circadian parameters under DD conditions, where rhythmicity is sustained only by the circadian clock, independent of environmental cues. Table 1 summarizes our findings in this approach.

**Table 1:** Summary of endogenous circadian parameters.

**Parameter**	**Sham**	**Implanted**	***p***
Period τ (hours)	23.58 ± 0.07	23.92 ± 0.06	<0.01
α (hours)	13.43 ± 0.90	13.08 ± 0.75	>0.05
Activity in α %	85.13 ± 1.95	83.31 ± 1.89	>0.05
α/ρ	1.31 ± 0.28	1.17 ± 0.13	>0.05
V index	0.69 ± 0.08	0.66 ± 0.04	>0.05
IS index	0.44 ± 0.03	0.31 ± 0.02	<0.01
IV index	0.68 ± 0.08	1.04 ± 0.1	<0.05

*Circadian rhythms of general activity in DD conditions. α, length of the subjective day; ρ, length of the subjective night. See methods for details on V, IS, and IV index calculation. Groups were compared by Student's t-test*.

**Figure 2:**
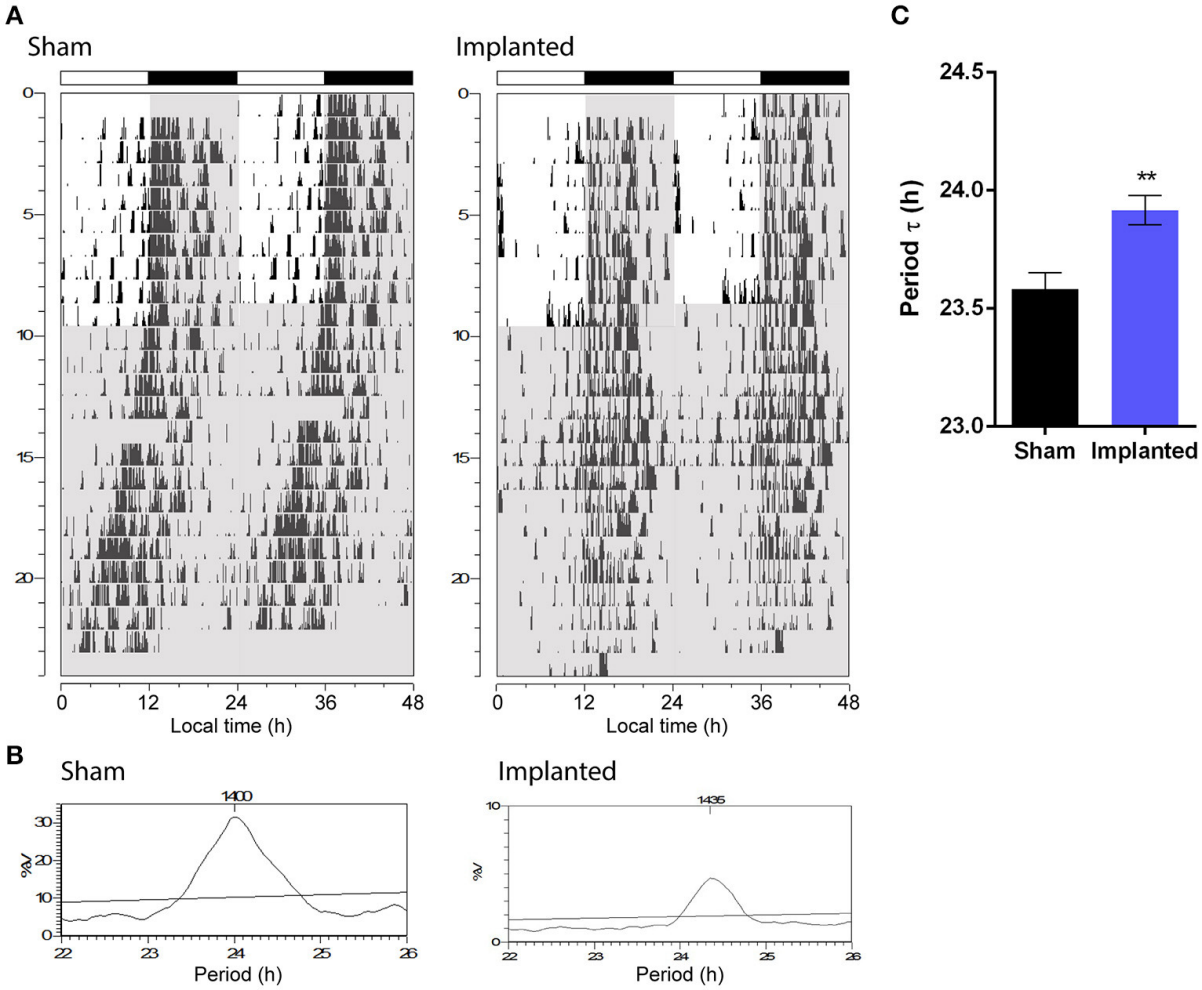
Hypothalamic glioma alters endogenous circadian timekeeping. **(A)** Representative actograms of control (sham) and implanted animals. Gray background indicates lights-off. **(B)** Representative Sokolove-Bushell's periodograms for sham and implanted animals showing the endogenous circadian period. **(C)** Hypothalamic tumors increase the endogenous circadian period (23.92 ± 0.06 h, *n* = 9 and 23.58 ± 0.07, *n* = 6, for implanted and control (sham) animals, respectively; ^**^p < 0.01, Student's *t*-test).

One of the most important parameters of the endogenous circadian rhythm is the period at which the clock runs without external cues. Animals implanted with hypothalamic gliomas presented a small, albeit significant, alteration in the endogenous period of the circadian clock, with larger τ values than sham controls (Figure 2; 23.92 ± 0.06 h, *n* = 9 and 23.58 ± 0.07, *n* = 6, for implanted and control animals, respectively; *p* < 0.01, Student's *t*-test).

**Figure 3:**
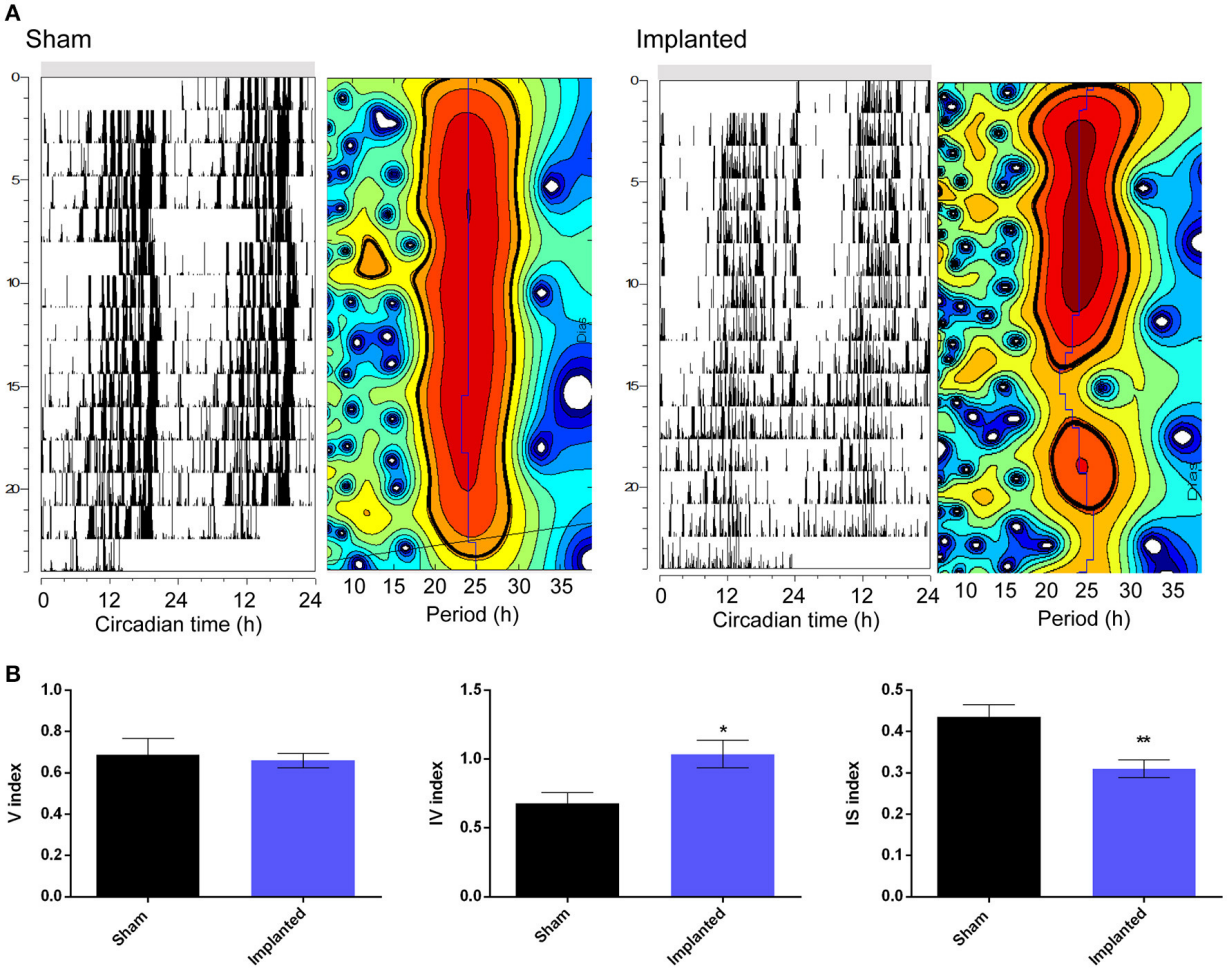
Hypothalamic gliomas affect endogenous period stability. **(A)** Representative actograms and their corresponding instantaneous period calculation of control (sham) and implanted animals in constant darkness conditions. For instant periods, heatmaps plots refer to the significance of the endogenous period calculated by wavelet analysis. The vertical axis represent the successive days of activity, with the same scale of the corresponding actograms. The horizontal axis indicates the period in hours. The blue line shows the period of maximum significance across days. Black lines delimitate periods that are statistically significant (Torrence and Compo, 1998). **(B)** Tumor-implanted animals showed higher fragmentation of locomotor activity rhythms, as well as less strength of coupling of the activity to the τ that best describes the rhythms, although no effect was found by analysis of τ variability through wavelet-based computations (described by IV, IS, and V indexes, respectively; Student's *t*-test, ^*^p < 0.05 for IV, ^**^p < 0.01 for IS and *p* > 0.05 for V indexes, *n* = 6 for control and *n* = 9 for implanted animals).

Besides affecting the endogenous period, we hypothesized that hypothalamic gliomas would alter the ability of the clock to sustain robust and stable endogenous rhythms. This hypothesis was assessed by analyzing activity consolidation during the subjective night and the presence of a stable τ throughout several days. We found no difference in the distribution of the activity, identified as α and ρ, during DD conditions between implanted and control animals (Table 1). However, examination of instant periods across the whole temporal series by means of wavelet analysis suggested that the endogenous period was less stable in implanted mice (Figure 3). Although analysis of τ variability through wavelet-based computations, using the V index, did not show statistically significant differences, a more stringent analysis through NPCRA demonstrated that implanted animals presented less robust rhythms under DD conditions. We calculated the IV index which is an indicator of the fragmentation of the locomotor activity, describing the duration and repetition of transition between rest and activity, and the IS index which quantifies the invariability between the days, that is, the strength of coupling of the activity to the τ that best describes the rhythms. We found that animals implanted with hypothalamic tumors showed weaker stability of the rhythm related to the coupling to a specific τ, and higher fragmentation of the activity pattern (Figure 3 and Table 1; for IS, *P* < 0.01; for IV *P* < 0.05; Student's *t*-test, *n* = 6 for controls, *n* = 9 for implanted mice).

### Effect of tumor implantation on synchronization to light-dark cycles

Besides alteration of endogenous timekeeping, hypothalamic gliomas might affect entrainment to environmental cues. In order to study the effects of hypothalamic gliomas in the ability of the circadian clock to synchronize to light-dark schedules, we analyzed the characteristics of activity rhythms both at a stable LD schedule and during resynchronization to an abrupt shift in the LD schedule (i.e., Jet Lag). The results of this set of experiments are summarized in Table 2. It should be noted that since animals were subjected to a Jet Lag protocol, the analysis was divided in three stages, before implantation (BI), post-implantation (PI) and after Jet Lag protocol (P-JL). For the P-JL stage, circadian parameters were only calculated after each animal reached full resynchronization to the new LD schedule.

**Table 2:** Summary of circadian parameters under LD conditions.

**Parameter**	**Group**	**BI**	**PI**	**P-JL**	***p*^a^**
Phase angle ψ (min)	Sham	−15.88 ± 5.6	−1.49 ± 3.43	3 ± 8.16	>0.05
	Implanted	−16.67 ± 17.3	33.66 ± 7.64	92.33 ± 15.28	<0.05
	*p*^b^	>0.05	>0.05	<0.001	
LD period (h)	Sham	24.08 ± 0.03	24.05 ± 0.02	24.05 ± 0.04	>0.05
	Implanted	23.99 ± 0.01	24.06 ± 0.02	24.06 ± 0.04	>0.05
	*p*^b^	>0.05	>0.05	>0.05	
Diurnal activity %	Sham	24.54 ± 1.91	26.73 ± 2.6	33.46 ± 2	>0.05
	Implanted	34.64 ± 2.22	34.00 ± 2.40	29.23 ± 2.98	>0.05
	*p*^b^	>0.05	>0.05	>0.05	
P_50_	Sham	2.78 ± 0.21			
	Implanted	3.96 ± 0.21			
	*p*^c^	<0.001			

*For phase angle, data was analyzed by repeated measures two-way ANOVA, with p < 0.05 for the interaction effect. Effects of treatment (tumor implantation) and stage were assessed by Holm-Sidak's multiple comparisons test. For LD period and diurnal activity percentage, data was analyzed by repeated measures two-way ANOVA with p > 0.05 for treatment, stage and interaction effects. For reentrainment rate, data was adjusted to a sigmoid dose-response curve with variable slope and differences between best-fit PS_50_ for Sham and Implanted groups were analyzed by extra sum-of-squares F-test*.

a *Post-test for stage factor*.

b *Post-test for treatment factor*.

c *Student's t-test*.

**Figure 4:**
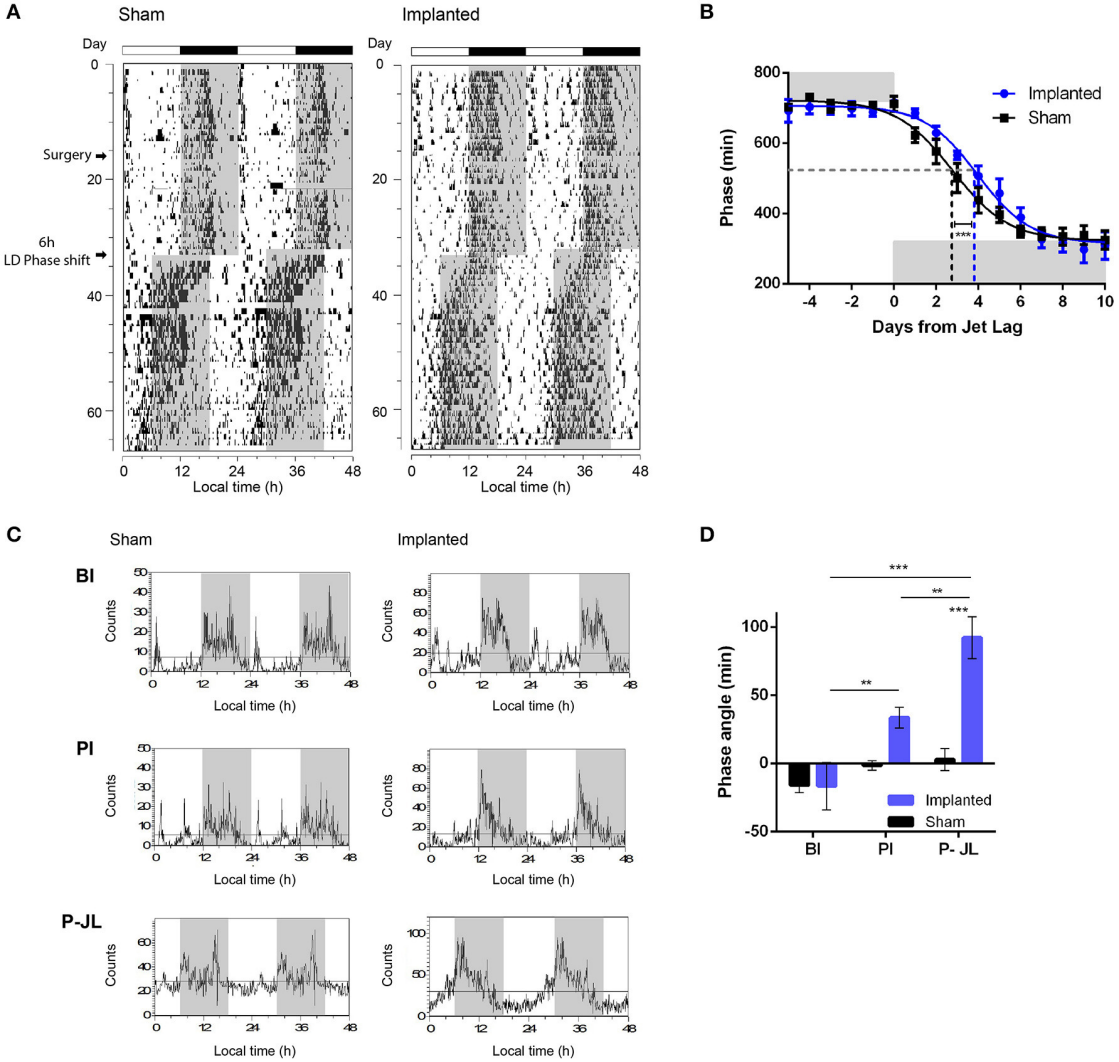
Synchronization to LD cycles is altered by hypothalamic gliomas. **(A)** Representative actograms of control (sham) and implanted animals under LD cycles and a simulated Jet-lag protocol (6-h advance in the LD cycle). **(B)** Tumor implantation resulted in slower resynchronization to abrupt shifts in the LD cycle. The phase of activity onset, in minutes, is plotted for each day before and after the Jet-lag protocol. Gray background indicates the time of lights-off. A continuous line indicates the adjusted sigmoid dose-response curve with variable slope for each group. The dashed gray line indicates the 50% phase resynchronization, and vertical dashed lines indicate the PS_50_ for each group. Control animals showed shorter PS_50_ adjusted value, indicating faster resynchronization (extra sum-of-squares *F*-test, ^***^p < 0.001, *n* = 6 for control and implanted animals). **(C)** Representative waveforms of general activity of control (sham) and implanted animals in three different stages of the protocol (BI, Before implantation; AI, After implantation; P-JL, Post Jet Lag). **(D)** Tumor-implanted animals exhibited an advanced phase angle between the circadian rhythm in general activity and the LD cycle. (repeated measures Two-Way ANOVA followed by Holm-Sidak's *post-hoc* test for stage and treatment-tumor implantation-factors, *p* < 0.05 for the interaction. Stage effects: ^**^p < 0.01 for implanted animals in PI vs. BI stage, ^**^p < 0.01 for implanted animals in P-JL vs. PI stage, and ^***^p < 0.001 for implanted animals in P-JL vs. BI stage, *p* > 0.05 for all Sham contrasts. Implantation effects: ^***^p < 0.001 for Sham vs. Implanted animals in the P-JL stage, *p* > 0.05 for all other contrasts; *n* = 6 for control and implanted animals).

We first characterized whether animals were able to effectively synchronize to the period imposed by an LD cycle. The period observed under LD conditions did not vary between control and implanted animals (Table 2), suggesting that hypothalamic gliomas do not impair the clock in terms of following an environmental cycle. Similarly, the amount of activity during the day did not vary significantly between implanted and control animals (Table 2). Next, when analyzing the rate of reentrainment to an abrupt phase change of the LD cycle, we found that implanted animals presented a slower resynchronization to a 6-h advance, with an average PS_50_ of 3.96 ± 0.21 days, as compared with the average PS_50_ of 2.78 ± 0.21 days for the control animals (Figures 4A,B and Table 2; extra sum-of-squares *F*-test between PS_50_ for Implanted and Sham mice, *p* < 0.001, *n* = 6 for control and implanted animals).This result points to an impairment in the ability of the circadian clock to adapt to changes in the Zeitgeber cycle in animals with hypothalamic gliomas.

Phase angle is defined as the difference between the phase of the circadian clock and the phase of its corresponding Zeitgeber, and is important in setting the relation between animal physiology and external environmental cues. We analyzed if the phase angle between the clock and the LD cycle was affected in animals bearing hypothalamic tumors. We found that implanted animals showed a clear advance in the phase angle between the activity onset and the time of light off, as compared with the sham controls, which became more pronounced with the progression of the tumor (Figure 4 and Table 2; repeated measures Two-Way ANOVA followed by Holm-Sidak's *post-hoc* test for stage and treatment factors, *p* < 0.05 for the interaction. Stage effects: *p* < 0.01 for implanted animals in PI vs. BI stage, *p* < 0.01 for implanted animals in P-JL vs. PI stage, and *p* < 0.001 for implanted animals in P-JL vs. BI stage, *p* > 0.05 for all Sham contrasts. Implantation effects: *p* < 0.001 for Sham vs. Implanted animals in the P-JL stage, *p* > 0.05 for all other contrasts; *n* = 6 for control and implanted animals). This finding indicates a persistent decline in the ability of the clock to set a proper phase relation with environmental cue as the hypothalamic tumor grows.

**Figure 5:**
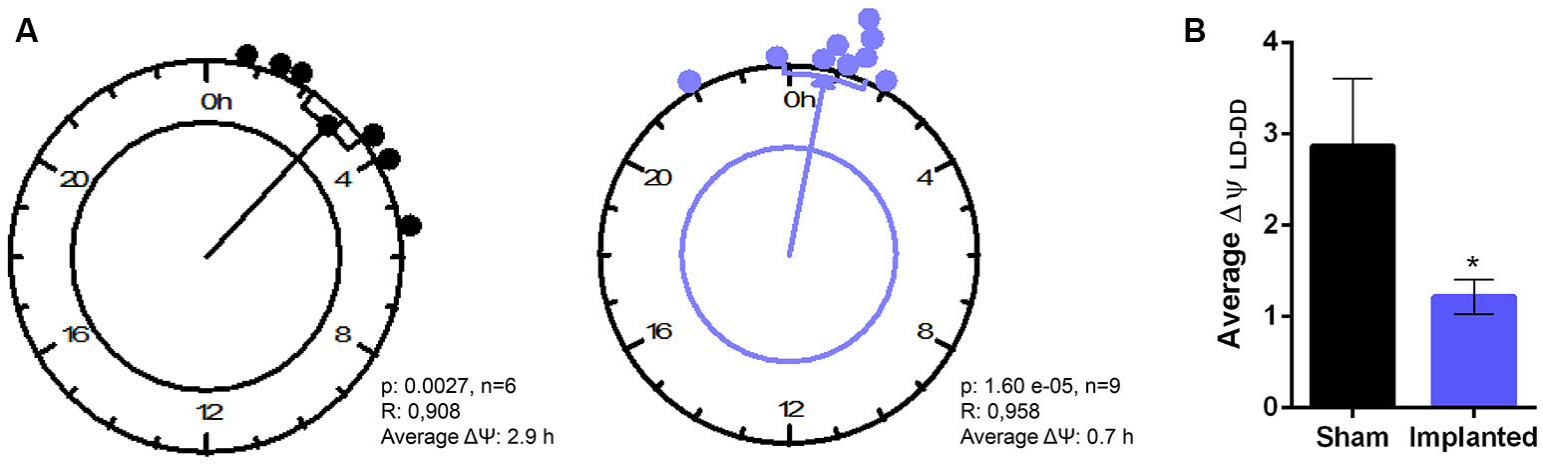
Hypothalamic gliomas alter the relation between clock outputs and LD cycles. **(A)** Rayleigh analysis of average phase differences between LD and DD conditions for individual animals. **(B)** Tumor implantation resulted in a smaller difference between the phase of general activity in LD and DD conditions (^*^p < 0.05 Student's *t*-test, *n* = 6 for controls, *n* = 9 for implanted).

Finally, to characterize more subtle effects of hypothalamic gliomas in the synchronization to LD cycles, we analyzed the difference between the phase of general activity during the first days of DD and compared it with the previous phase during LD conditions. If an animal is properly synchronized to the LD schedule, it can be expected that the phase in LD conditions represents a good predictor of the phase once the animal is transferred to constant DD conditions. We calculated the individual phase difference between the average phase both in LD and in DD conditions and, surprisingly, we found that sham animals presented a bigger phase difference than animals carrying hypothalamic gliomas (Figure 5, *p* < 0.05, Student's *t*-test, *n* = 6 for controls, *n* = 9 for implanted mice).

## Discussion

The mammalian circadian clock is able to generate a coherent output through a complex combination of molecular and network mechanisms that can be synchronized by light inputs. Since pathologies affecting hypothalamic function can disrupt circadian timekeeping (Giustina and Braunstein, 2016), the presence of anomalous cells, such as glia-derived tumor cells could then modify circadian output clock, and be of relevance in the diagnosis of this tumors. Therefore, we aimed to study the effects of hypothalamic tumors on the circadian system, using an animal model suitable both for human tumor implantation and characterization of circadian parameters (Paladino et al., 2013).

The implantation of intracranial gliomas in the suprachiasmatic hypothalamic region resulted in both alteration of endogenous circadian timekeeping and in subtle changes in the ability of the clock to synchronize to environmental cycles. These phenomena should be interpreted in an integrative way, since the synchronization process also depends on the endogenous characteristics of the oscillator. Our results point to three important alterations in animals carrying hypothalamic gliomas: longer and unstable endogenous circadian period, alteration in the phase relation between the clock and the Zeitgeber, and a possible decrease of masking mechanisms.

The endogenous period of the circadian clock depends on individual molecular oscillations that occur in each cell, as well as on the coupling between different cells and regions of the SCN (Evans, 2016). The pace of the molecular clock can be modulated by the alteration of the transcription of clock genes, or by post-transcriptional or post-translational modifications on the products of the genes, affecting their stability or subcellular localization (Lowrey and Takahashi, 2011). Gliomas impose a deep alteration on the environment where they develop, secreting a variety of factors, increasing the vascularization, and remodeling the extracellular matrix, among others. Within the molecules that are increased in the tumor microenvironment, glutamate and immune factors such as TNF-α, IL-1α, and CCL2 should be taken into special consideration. Glioma cells increases extracellular glutamate levels both by means of glutamate release and by silencing of EAAT genes; indeed, increases in glutamate levels are proposed to favor neuronal death through excitotoxicity generating a physical niche for tumor growth (Watkins and Sontheimer, 2012). Glutamate is released in the SCN in response to light, activating a signaling cascade that induces clock gene expression (Golombek and Rosenstein, 2010). High glutamate levels in DD conditions could modulate clock gene expression through signaling pathways normally activated by light, inducing the alterations in τ found in implanted animals. Regarding the alteration of immune-related signaling molecules, TNF-α, IL-1β, and CCL2 have been shown to modulate the circadian clock, at the SCN level (Leone et al., 2012; Duhart et al., 2016). These, and/or other cytokines/chemokines, which are increased in the tumor microenvironment (Christofides et al., 2015; Vakilian et al., 2017) could modulate either the pace of the molecular clock or the coupling among different neurons.

Our results also showed that the proper phase relation between the oscillator outputs and the environmental cues is impaired in animals carrying hypothalamic gliomas, as evidenced by the increased phase angle between general activity rhythm onset and the time of lights-off. The alterations in the characteristics of the clock synchronization to LD cycles could be affected by the same mechanisms that lead to changes in endogenous pacemaking, which could also alter the shape and amplitude of the phase response curve (i.e., the differential effect of light on the phase of circadian rhythms according to the time of stimulation). In particular, abnormal glutamate levels in the SCN could affect synchronization to light, since this neurotransmitter is normally released preferentially during the day in this region, and induces phases adjustments if released during the night (Gillette and Mitchell, 2002). Also, TGF-α is elevated in the glioma microenvironment (Schlegel et al., 1990), and this molecule has been described to mediate the relation between the SCN and different clock outputs (Kramer et al., 2001). A dysregulation of TGF-α at the SCN level could thus lead to alterations in the coupling between the clock and the control of activity output.

Circadian entrainment was also evaluated by comparing the differences between the phase of general activity in LD and in the first days of DD conditions (Figure 5). If the oscillator is truly synchronized to the LD cycle, the phase during the first days in DD is predicted to be similar to the previous phase under LD. We found that control animals showed a bigger difference between the phase in LD and in DD conditions, which could be interpreted as a signal of a masking phenomenon in which the output (general activity) is directly modulated by the Zeitgeber. Masking might serve as a fine adjustment mechanism in the synchronization process; although the specific anatomical structure sustaining this phenomenon has not been identified. It has been has been suggested that masking might rely on TGF-α acting on the ventral subparaventricular zone (vSPZ, a region adjacent to the SCN) of the hypothalamus (Kramer et al., 2001). As stated before, TGF-α has been detected in human glioblastomas, correlating with the proliferative aggressiveness of the tumors (von Bossanyi et al., 1998). Alteration of this signal in the hypothalamic tumor microenvironment could impair the mechanism by which masking modulates activity rhythms, and this could be evidenced in the smaller phase difference between LD and DD conditions in animal bearing hypothalamic gliomas.

It should also be noted that gliomas have a strong influence in the physiology of the normal glial cells compromised in the tumor microenvironment. Astrocytes have recently been shown to be active players in setting the endogenous timekeeping of the circadian clock (Brancaccio et al., 2017; Tso et al., 2017). In addition, activation of these cells by immune factors induces changes in their molecular clock and, in response, astrocytes can modulate clock gene expression in the SCN (Duhart et al., 2013a). Gliomas have been shown to alter the coupling between astrocytes and the vasculature (Watkins et al., 2014), and to render astrocytes in an activated state (Lee et al., 2011; Raore et al., 2011). These or other changes in astroglial functioning could have an impact in the circadian clock, contributing to the effects seen in animals bearing hypothalamic tumors.

In summary, the glioma microenvironment greatly alters the levels of different molecules that can modulate clock gene expression, activate the photic synchronization pathway, and/or mediate the mechanisms underlying masking to light. These phenomena were evidenced in our murine model of hypothalamic gliomas. While the effects of gliomas located in the hypothalamic/optic tract region on circadian rhythms have not been extensively analyzed in patients yet, one report from an hypothalamic astrocytoma case compromising the SCN region described, among other symptoms, an inversion in the wake-sleep cycle (Haugh and Markesbery, 1983). Also, pediatric patients carrying craniopharyngiomas, which usually compromises the optic tract and produce injury in the hypothalamus, present daytime sleepiness and alterations in the melatonin secretion rhythm (Müller et al., 2002). Recently, adult patients suffering from craniopharyngiomas were also described to present impaired 24 h sleep-wake and temperature circadian rhythms (Foschi et al., 2017). It has been previously reported that sleep disturbances are related to high-grade glioma progression (Yavas et al., 2012), although the availability of data regarding the effects of solid brain tumors on sleep/wake cycle is still limited (Armstrong et al., 2017). Our work shows that hypothalamic gliomas can alter the endogenous properties of the clock, as well as the relation between the pacemaker, environment and output variables, suggesting that assessing simple circadian parameters, such as body core temperature, as well as sleep/wake cycle analysis, might be of importance during the clinical assessment and track of this disease. Further research in the characterization of the influence of hypothalamic/optic tract tumors on circadian rhythms could also be of importance in developing new factors or parameters that help both diagnosis and monitoring the progression of these diseases.

## Author contributions

JD and LB helped in the design of the research, performed all experiments and analyzed data. LM and CC helped in technical aspects of the research, as well as in the analysis. DG supervised all research. All authors co-wrote the paper.

### Conflict of interest statement

The authors declare that the research was conducted in the absence of any commercial or financial relationships that could be construed as a potential conflict of interest.
